# The Comparison of Stereotactic Body Radiation Therapy and Intensity-Modulated Radiation Therapy for Prostate Cancer by NCCN Risk Groups

**DOI:** 10.3389/fonc.2016.00184

**Published:** 2016-08-23

**Authors:** Anthony Ricco, Genevieve Manahan, Rachelle Lanciano, Alexandra Hanlon, Jun Yang, Stephen Arrigo, John Lamond, Jing Feng, Michael Mooreville, Bruce Garber, Luther Brady

**Affiliations:** ^1^Philadelphia Cyberknife, Delaware County Memorial Hospital, Havertown, PA, USA; ^2^Drexel University College of Medicine, Philadelphia, PA, USA; ^3^University of Pennsylvania, Philadelphia, PA, USA

**Keywords:** IMRT, SBRT, prostate cancer, NCCN guidelines, freedom from biochemical failure, toxicity

## Abstract

**Objectives:**

The primary objective of this study is to compare freedom from biochemical failure (FFBF) between stereotactic body radiation therapy (SBRT) and intensity-modulated radiation therapy (IMRT) for patients with organ confined prostate cancer treated between 2007 through 2012 utilizing the 2015 National Comprehensive Cancer Network (NCCN) risk stratification guidelines. A secondary objective is to compare our updated toxicity at last follow-up compared with pretreatment with respect to bowel, bladder, sexual functioning, and need for invasive procedures between the two groups.

**Methods:**

We retrospectively reviewed 270 consecutive men treated with either SBRT (*n* = 150) or IMRT (*n* = 120) at a community hospital with two distinct radiation departments and referral patterns. Charts were reviewed for pretreatment and treatment factors including race, age, clinical T stage, initial PSA, Gleason score, use of androgen deprivation therapy, treatment with SBRT vs. IMRT, as well as stratification by 2015 NCCN guidelines. Kaplan–Meier (KM) methodology was used to estimate FFBF, with statistical comparisons accomplished using log rank tests. Multivariable Cox proportional hazard modeling was used to establish independent factors prognostic of biochemical failure. Descriptive statistics were used to describe toxicity graded by a modified Radiation Therapy Oncology Group (RTOG) late radiation morbidity scoring system.

**Results:**

Significant prognostic factors in univariate analysis for FFBF included NCCN risk groups (*p* = 0.0032), grade (*p* = 0.019), and PSA (*p* = 0.008). There was no significant difference in FFBF between SBRT vs. IMRT (*p* = 0.46) with 6-year actuarial FFBF of 91.9% for SBRT and 88.9% for IMRT. Multivariable analysis revealed only the NCCN risk stratification to be significant predictor for FFBF (*p* = 0.04). Four-year actuarial FFBF by NCCN risk stratification was 100% very low risk, 100% low risk, 96.5% intermediate risk, 94.5% high risk, and 72.7% very high risk. There were no grade 3 gastrointestinal or genitourinary toxicities for either SBRT or IMRT at last follow-up.

**Conclusion:**

No significant difference in FFBF was found between SBRT and IMRT for organ confined prostate cancer in multivariable analysis within this retrospective data set. Overall toxicity was low. The 2015 NCCN risk stratification was validated in this population and was the only significant factor for FFBF in multivariable analysis.

## Introduction

Intensity-modulated radiation therapy (IMRT) has been a standard radiation modality used in the treatment of organ confined prostate cancer. Ten-year actuarial data (median follow-up of 8 years) is available for high dose IMRT up to 81 Gy which demonstrates high efficacy in preventing biochemical failure with acceptable side effect rates (1). Stereotactic body radiation therapy (SBRT) has been accepted as an “appropriate alternative for select patients with low to intermediate risk disease” as per the American Society for Radiation Oncology (ASTRO) policy update of April 2013 and is also supported by the National Comprehensive Cancer Network (NCCN). SBRT publications have validated freedom from biochemical failure (FFBF) and side effect rates comparable to IMRT (2, 3).

The combination of prostate cancer’s low alpha/beta ratio, known benefit of dose escalation, and efficacy/safety of high dose rate brachytherapy led to single institutional, multi-institutional, and randomized clinical trials of SBRT for the treatment of prostate cancer (4, 5). Despite a clear cost savings for SBRT compared with IMRT in a recent pooled Medicare database analysis, a 7.5% absolute increased risk of genitourinary (GU) toxicity at 24 months was associated with SBRT (6). To date, the only direct head-to-head comparison between the two modalities with respect to FFBF and toxicity in a single database is from our institution reporting 5-year propensity score analysis with no significant difference between IMRT and SBRT noted (7).

The primary goal of this study is to update FFBF between SBRT and IMRT for men with organ confined prostate cancer treated between 2007 through 2012 utilizing the 2015 NCCN risk stratification guidelines in a larger database from our institution. A secondary objective is to confirm prognostic factors in this larger data base through multivariable analysis. An additional objective is to evaluate toxicity at last follow-up compared with pretreatment with respect to bowel, bladder, sexual functioning, and need for invasive procedures in our updated database with comparisons between IMRT and SBRT.

## Materials and Methods

Two hundred seventy consecutive men treated for organ confined prostate cancer using either SBRT (*n* = 150) or IMRT (*n* = 120) between 2007 through 2012 were reviewed on this IRB approved retrospective study. Patients were treated with SBRT at an outpatient radiation facility and IMRT at the community hospital site, both under the same license and radiation department but with distinct geographic and referral patterns. PSA nadir was defined as the lowest PSA value following SBRT or IMRT. Biochemical failure was assessed using the Phoenix Nadir + 2 definition (8). Toxicity was assessed using the Radiation Therapy Oncology Group (RTOG) group criteria with modification. If patients required medication for treatment of lower urinary tract symptoms before radiation or at last follow-up, our modification of the RTOG system placed them into grade 2, which is not required in the RTOG late toxicity scoring system. Erectile function (EF) was graded as grade 0: no issues achieving erection adequate for intercourse, grade 1: erections sufficient for intercourse most of the time but reduced from baseline, grade 2: requiring medications for attainment of EF adequate for intercourse, and grade 3: erections not sufficient for intercourse most of the time despite use of oral medications or can achieve erections with implanted device.

Stereotactic body radiation therapy was delivered using the Cyberknife system with multi-plan inverse treatment planning and motion tracking of internal fiducials. Treatment planning began with transrectal or transperineal ultrasound-guided placement of four gold fiducials into the prostate. A CT scan was obtained 10–14 days later to allow inflammation to subside and ensure fiducials did not migrate. T2 fast echo MRI was obtained and three-dimensionally registered by fiducials to the CT. All patients were simulated in the supine position with immobilization, full bladder, and empty rectum. The clinical target volume (CTV) was the prostate for very low- and low-risk patients, the prostate plus the proximal seminal vesicle for most intermediate, high, and very high-risk patients. Pelvic lymph nodes were never targeted. Five fractions were prescribed to the planning target volume (PTV) that consisted of the CTV with a 5-mm margin in all directions except 3 mm posteriorly. Dose administered was standard throughout our center, which was based on in-house protocol and clinical trial participation. Initially, our first few patients were treated with 35 Gy (EQD2 = 85, assuming an alpha/beta of 1.5 for prostate cancer), followed by 37.5 Gy (EQD2 = 96.4), and at the time of this publication 36.25 Gy (EQD2 = 90.6), which began 2008 when we participated in a national protocol. The dosimetric goal was to cover at least 95% of the PTV with the prescribed dose normalized to the 75–85% isodose line (dose heterogeneity 17–33%). Less than 1 cm^3^ of rectum received 36 Gy, 50% of the prescribed dose could not cross the posterior rectal wall, and <10 cm^3^ of bladder received 37 Gy. These dose constraints were utilized for each prostate dose level. The average/median CTV and PTV were 56.7/50.1 cc (SD = 25.7 cc) and 97.9/89.9 cc (SD = 37.9 cc), respectively. Orthogonal 120-kV X-ray image pairs were obtained throughout treatment for use in motion tracking. The real-time prostate position was locked-on by the relative fiducial position on the X-rays. For those patients with evenly distributed fiducials in the prostate quadrants, the prostate’s rotation was also tracked and corrections were made in real time.

For IMRT, the PTV was defined as the prostate with 8 mm margin in all directions except 5 mm posteriorly in very low- and low-risk patients, prostate plus seminal vesicles with 5/8 mm margin for intermediate and some high risk patients, and prostate plus seminal vesicles plus true pelvic lymph nodes with 5/8 mm margin for most high and very high risk patients. Dose constraints to the rectum were defined as V65 < 17% and V40 < 35%, while the bladder constraints were V65 < 25% and V40 < 50%. All patients were simulated in the supine position with immobilization, full bladder, and empty rectum. CT and MRI treatment planning was completed with merging of the images for contouring of the prostate. Image guidance was provided by ultrasound pretreatment daily, and patients were treated with full bladder daily. Usually, five to seven isocentric beams were utilized to treat the prostate with 6 MV photons optimized with an inverse optimization algorithm with at least 95% of the prostate receiving the prescribed dose. Approximately 28% of men were treated to the prostate alone, 28% to the prostate + seminal vesicles, and 44% to the true pelvic lymph nodes + seminal vesicles + prostate. The majority of patients received 75.6 Gy (EQ2 = 71.3) to the prostate in 1.8 Gy per fraction (72 to <75.6 Gy = 16.8%, 75.6 Gy = 60.5%, and >75.6 Gy = 22.7%).

Kaplan–Meier (KM) methodology was used to estimate FFBF from end of radiation treatment (EOT), with statistical comparisons accomplished using log rank tests. Simple Cox proportional hazard modeling was used to estimate hazard ratios; multivariable modeling was used to establish independent factors prognostic of biochemical failure among NCCN risk groups and treatment group (IMRT vs. SBRT). Additionally, multivariable modeling was performed using Gleason Score, pretreatment PSA, and treatment group. Separate multivariable models were examined to avoid multicollinearity issues. All analyses were accomplished using SAS V9.4 (Cary, NC).

## Results

### Patient and Treatment Characteristics

The median follow-up time for all patients was 50.2 months (mean = 55.31, range of 4–108 months). The median follow-up time for SBRT and IMRT patients was 45.53 and 53.38 months, respectively.

Pretreatment and treatment factors are described in Table 1. Patients were assigned retrospectively by risk groups based on the 2015 NCCN risk stratification guidelines. More patients had PSA < 10, Gleason score 6 or 7, and T1c/T2a clinical stage. The median age for IMRT patients was 72 years, whereas the median age for SBRT patients was 67 years. There is a statistically significant difference in distribution of patient and treatment characteristics by radiation treatment group with regard to age, race, T stage, Gleason score, NCCN risk groups, and use of androgen deprivation therapy (ADT), but not PSA.

**Table 1:** **Patient descriptive statistics**.

Pt characteristics	SBRT		IMRT		*p*-Value*
**Number of Pts**.	150		120		
**Age at diagnosis**	**Years**		**Years**		**<0.0001**
Median (IQR)	67 (11.8)		72 (9.2)		
Range	44–88		56–89		
**Pre-tx PSA**	**ng/mL**		**ng/mL**		**0.1403**
Mean (SD)	8.1 (7.8)		10.8 (18.7)		
Median (IQR)	6 (3.7)		6.2 (4.5)		
**Pre-tx PSA**	**Number of Pts**.	**Percent**	**Number of Pts**.	**Percent**	**0.2644**
<10 ng/mL	122	81.0	92	76.7	
10–20 ng/mL	19	13.0	14	11.7	
>20 ng/mL	9	6.0	14	11.7	
**T-stage**	**Number of Pts**.	**Percent**	**Number of Pts**.	**Percent**	**<0.0001**
T1c	113	75.3	43	35.8	
T2a	20	13.3	22	18.3	
>T2a	17	11.3	55	45.8	
**Gleason score**	**Number of Pts**.	**Percent**	**Number of Pts**.	**Percent**	**<0.0001**
5–6	83	55.3	34	28.3	
7	55	36.7	61	50.8	
8+	12	8.0	25	20.8	
**NCCN 2015 risk group**	**Number of Pts**.	**Percent**	**Number of Pts**.	**Percent**	**<0.0001**
Very low	33	22.0	9	7.5	
Low	34	22.7	13	10.8	
Intermediate	51	34.0	38	31.7	
High	25	16.7	48	40.0	
Very high	7	4.6	12	10.0	
**Race *N* = 145**	**Number of Pts**.	**Percent**	**Number of Pts**.	**Percent**	**<0.0001**
African-American	49	32.7	17	14.2	
Caucasian	90	60.0	101	84.2	
Other	7	4.7	1	0.8	
Unknown	4	2.7	1	0.8	
**Hormone treatment**	**Number of Pts**.	**Percent**	**Number of Pts**.	**Percent**	**<0.0001**
noADT	109	73	34	28	
ADT	41	27	86	72	

*Continuous variable p-value computed by t-test. Categorical variable p-value computed by fisher exact test.

Of those patients treated with SBRT, 80% were treated with either 35 or 36.25 Gy considered the “low dose” group, whereas 20% were treated with 37.5 Gy considered the “high dose” group. Our previous publication described the effect of higher SBRT dose on outcome and will not be further discussed here (9).

### PSA and Biochemical Control

**Figure 1:**
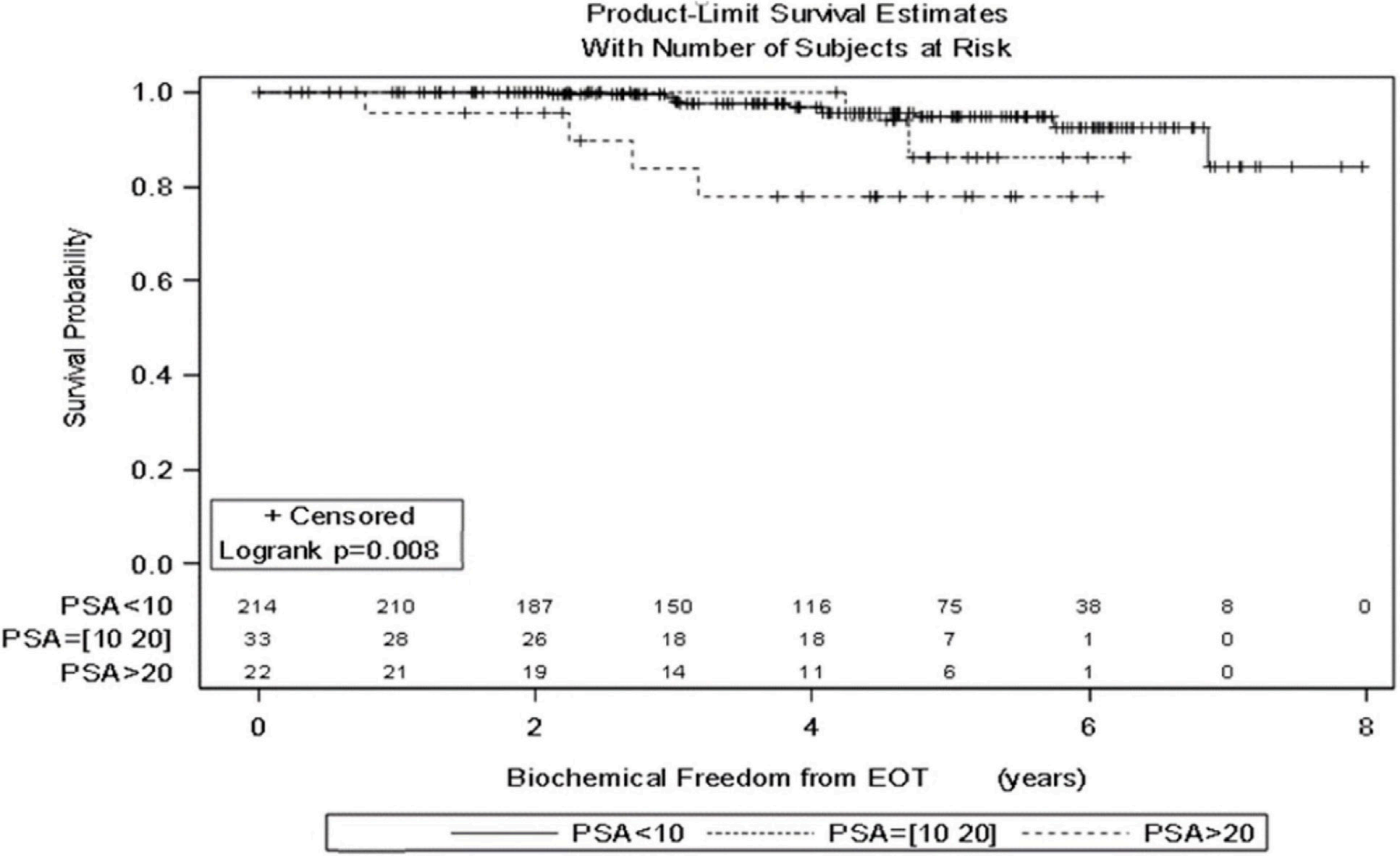
**KM curve stratifying FFBF by Initial PSA**.

**Figure 2:**
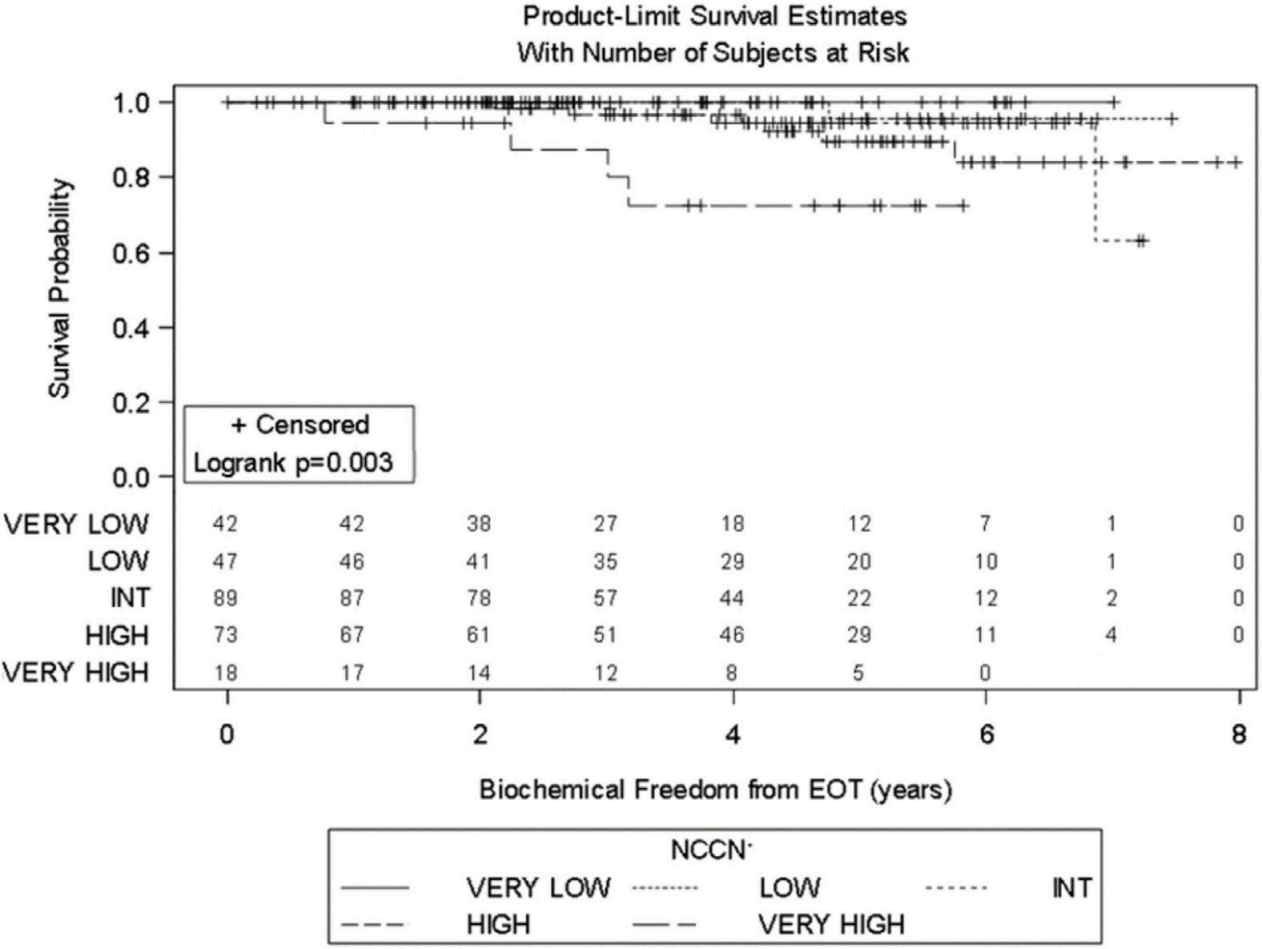
**KM curve stratifying FFBF by 2015 NCCN Guidelines**.

**Figure 3:**
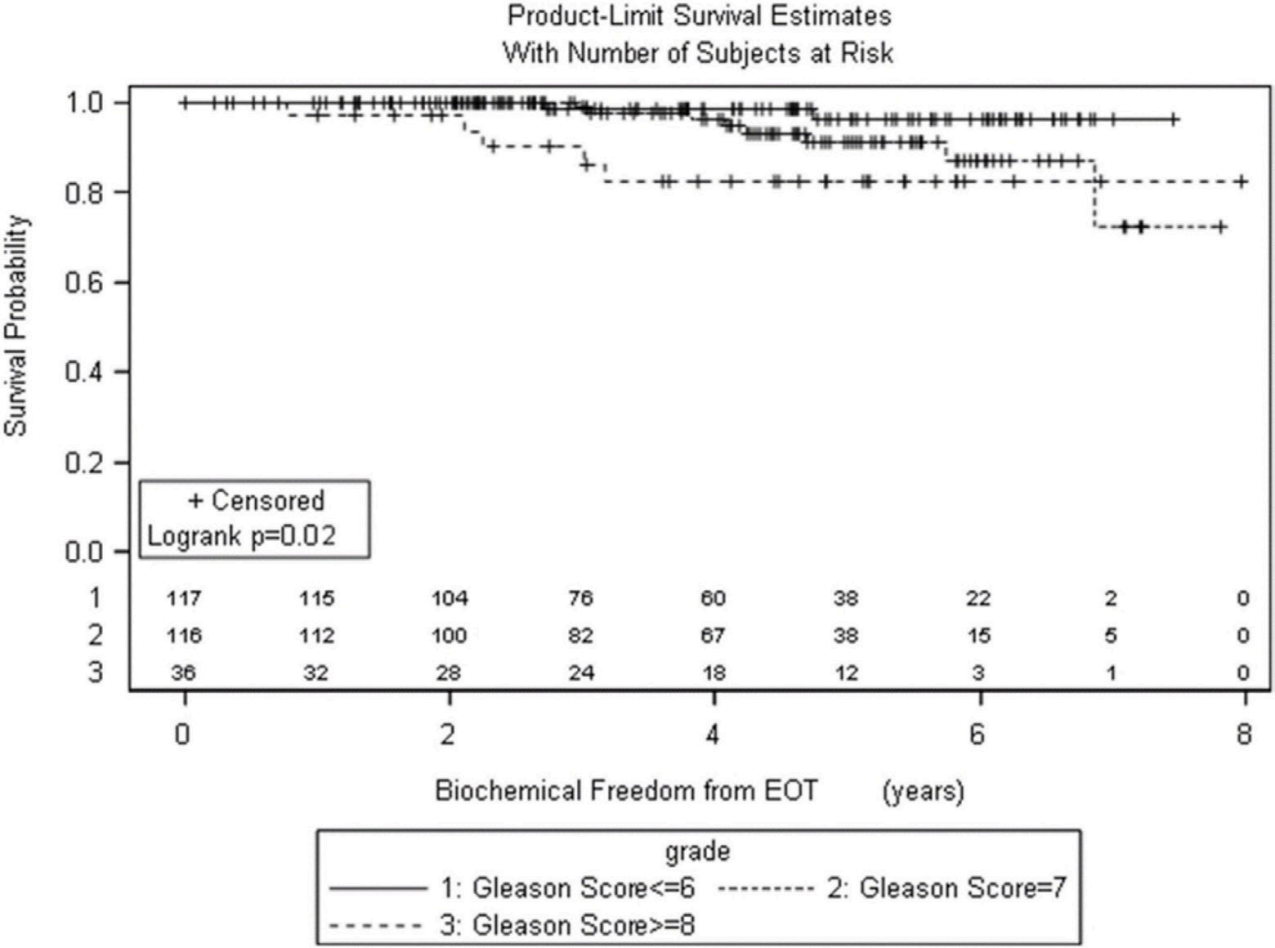
**KM curve stratifying FFBF by Gleason score**.

Significant prognostic factors in univariate analysis for FFBF included initial PSA (*p* = 0.008), NCCN risk groups (*p* = 0.003), and Gleason score (*p* = 0.02) (Figures 1–3). No significant differences in FFBF were observed by age, T-stage, race or use of androgen deprivation.

**Figure 4:**
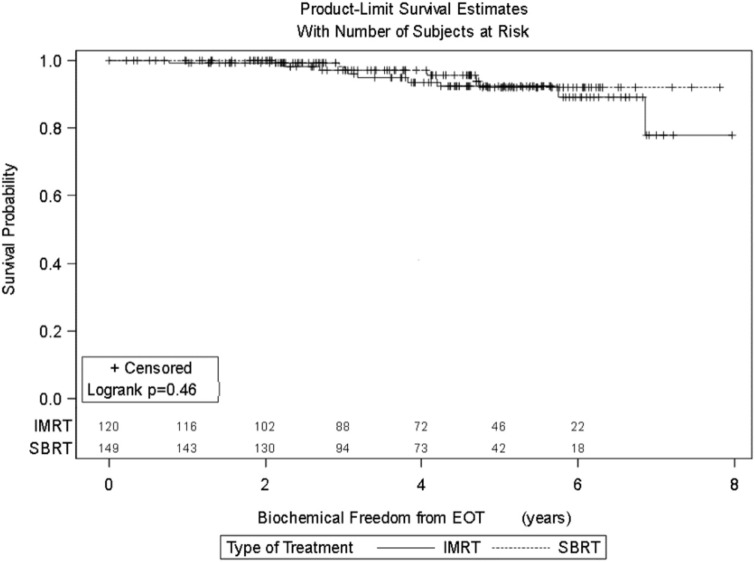
**KM curve stratifying FFBF by treatment type (IMRT vs. SBRT)**.

Four-year actuarial FFBF by NCCN risk stratification was 100% very low risk, 100% low risk, 96.5% intermediate risk, 94.5% high risk, and 72.7% very high risk.^1^ Four-year actuarial FFBF rates were 96.7% for PSA < 10, 100% for PSA 10–20 and 77.9% for PSA > 20. Five-year actuarial FFBF rates stratified by Gleason score were GS ≤ 6 96.5%, GS = 7 91.1%, and GS ≥ 8 82.4%. Six-year actuarial FFBF was 91.9% for the SBRT group and 88.9% for the IMRT group with no significant difference in univariate analysis (*p* = 0.46) (Figure 4; Table 2).

**Table 2:** **Univariate analysis of FFBF by multiple variables**.

		1-year (%)	2-year (%)	3-year (%)	4-year (%)	5-year (%)	*p*-Value
All patients	*n* = 270	99.6	99.6	97.7	95.4	92.2	
Age	≤69^a^	99.3	99.3	95.5	94.5	93.2	0.8337
	>69^a^	100.0	100.0	100.0	96.4	91.2	
Treatment	SBRT	100.0	100.0	98.2	97.1	92.0	0.4608
	IMRT	99.2	99.2	97.1	93.6	92.2	
Risk group	Very low	100.0	100.0	100.0	100.0	100.0	0.0032
	Low	100.0	100.0	100.0	100.0	95.7	
	Int.	100.0	100.0	98.3	96.5	94.2	
	High	100.0	100.0	96.5	94.5	89.7	
	Very high	94.4	94.4	87.2	72.7	72.7	
Pre-tx PSA	<10	100.0	100.0	98.8	96.7	94.6	0.0083
	10–20	100.0	100.0	100.0	100.0	86.3	
	>20	95.5	95.5	83.9	77.9	77.9	
T-stage	T1c/T2a	100.0	100.0	99.3	97.0	93.0	0.1184
	T2B/T2C	100.0	100.0	94.4	92.2	89.8	
	T3a	83.3	83.3	83.3	83.3	.	
Gleason score	5–6	100.0	100.0	98.8	98.8	96.5	0.0199
	7	100.0	100.0	98.8	96.2	91.1	
	8+	97.1	97.1	90.1	82.4	82.4	
ADT	No ADT	100.0	100.0	98.1	98.1	92.4	
	ADT	99.2	99.2	97.2	92.8	91.4	0.2394

*^a^Median*.

Seven patients treated with SBRT experienced biochemical failure, one in the low-risk group, two each in the intermediate, high, and very high-risk groups, all from the low dose category. Nine patients treated with IMRT experienced biochemical failure, two in the intermediate-risk group, four in the high-risk group, and three in the very-high risk groups.

Initial multivariable analysis included the NCCN risk groups as well as radiation treatment modality (IMRT vs. SBRT). Because Gleason score and PSA are a component of the NCCN risk groups, a second multivariable analysis included only Gleason score, PSA, and radiation treatment modality to avoid inappropriate weighing of prognostic factors. Only NCCN risk stratification was a significant predictor for FFBR in multivariable analysis (*p* = 0.04).

### Toxicity

Five patients (3.3%) experienced acute grade 3 GU toxicities that resolved to either grade 1 or grade 2 by last follow-up in the SBRT group. Three of the five patients required TURP for resolution of acute symptoms, all three received 37.5 Gy.

**Figure 5:**
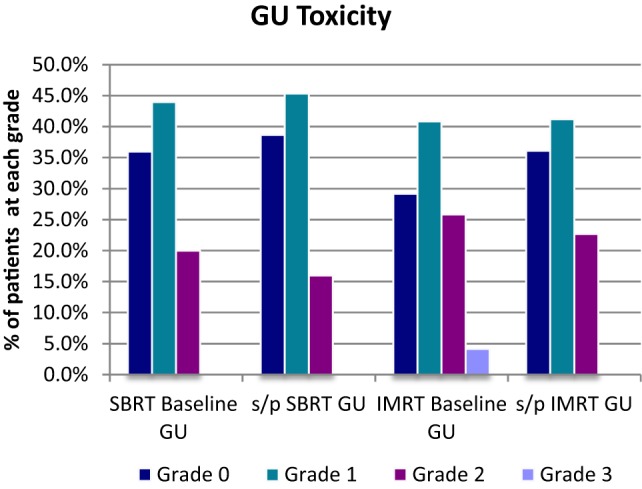
**GU toxicity**.

For the SBRT group at most recent follow-up, GU toxicities were grade 2 in 16% compared with 20% grade 2 at baseline before treatment. Of the 16% grade 2 patients at last follow-up, 18/24 were graded as such purely due to a requirement for medication for lower urinary tract symptoms. Of the remaining six patients, three had mild hematuria with one patient on warfarin and one patient on finasteride. Two patients had incontinence, and one patient required self-catheterization. There were no late grade 3 GU toxicities following SBRT at most recent follow-up (Table 2; Figure 5).

For the SBRT group at most recent follow-up, gastrointestinal (GI) toxicities were grade 2 in 2.7% compared with 1.3% grade 2 at baseline before treatment. No late grade 3 GI toxicity was reported following SBRT.

**Figure 6:**
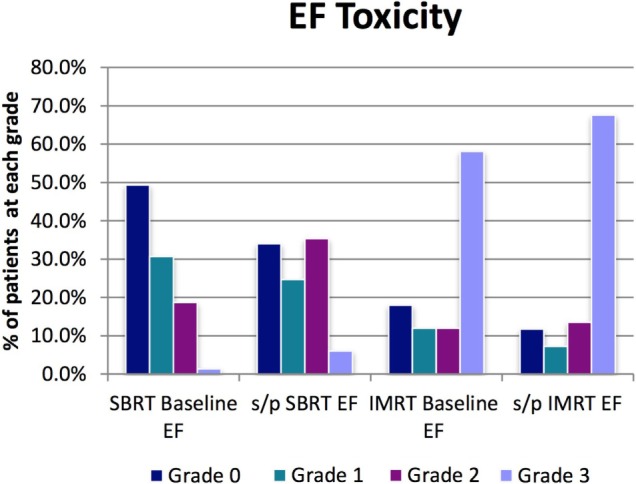
**EF toxicity**.

For the SBRT group at most recent follow-up, EF was grade 2 (requiring medication for adequate EF) in 35%, compared with 18.7% at baseline before treatment. Six percent of men at most recent follow-up had EF inadequate for intercourse despite medication or required penile prosthesis (grade 3) compared with 1.3% grade 3 at baseline before treatment. Ninety-seven percent of men with EF adequate for intercourse at presentation without medications (grades 0–1) were still potent either with or without medication at last follow-up (grades 0–2) (Table 3; Figure 6).

**Table 3:** **Baseline profiles (GU, GI, and EF) for SBRT and IMRT and toxicity profile at last FU**.

GU	SBRT *n* = 150	*n* = 150	IMRT *n* = 120	*n* = 119
Grade	SBRT baseline GU	s/p SBRT GU	IMRT baseline GU	s/p IMRT GU
0	36.0%	38.7%	29.2%	36.1%
1	44.0%	45.3%	40.8%	41.2%
2	20.0%	16.0%	25.8%	22.7%
3	0.0%	0.0%	4.2%	0.0%
*n*=	150	150	120	119

**GI**	**SBRT *n* = 150**	***n* = 150**	**IMRT *n* = 120**	***n* = 119**
**Grade**	**SBRT baseline GI**	**s/p SBRT GI**	**IMRT baseline GI**	**s/p IMRT GI**

0	87.3%	88.7%	93.3%	90.8%
1	11.3%	8.7%	5.0%	6.7%
2	1.3%	2.7%	1.7%	2.5%
3	0.0%	0.0%	0.0%	0.0%
*n*=	150	150	120	119

**EF**	**SBRT *n* = 150**	***n* = 150**	**IMRT *n* = 117**	***n* = 111**
**Grade**	**SBRT baseline EF**	**s/p SBRT EF**	**IMRT baseline EF**	**s/p IMRT EF**

0	49.3%	34.0%	17.9%	11.7%
1	30.7%	24.7%	12.0%	7.2%
2	18.7%	35.3%	12.0%	13.5%
3	1.3%	6.0%	58.1%	67.6%
*n*=	150	150	117	111

For the IMRT group at most recent follow-up, GU toxicities were grade 2 in 23% compared with 26% grade 2 at baseline before treatment. Of the 23% grade 2 patients at last follow-up, 20/27 were graded as such purely due to a requirement for medication for lower urinary tract symptoms. Of the seven remaining IMRT patients with grade 2 toxicity, both hematuria cases were confounded with use of anticoagulant, with five other grade 2 toxicity cases requiring cystoscopy or other forms of invasive diagnostic studies. There were no late grade 3 GU toxicities following IMRT, which improved from 4.2% at baseline before treatment (Table 3; Figure 5).

For the IMRT group at most recent follow-up, GI toxicities were grade 2 in 2.5% compared with 1.7% grade 2 at baseline before treatment. No late grade 3 GI toxicities were reported following IMRT.

For the IMRT group at most recent follow-up, EF was grade 2 (requiring medication for adequate EF) in 14%, compared with 12% at baseline before treatment. Sixty-eight percent of men at most recent follow-up had EF inadequate for intercourse despite medication or required penile prosthesis (grade 3) compared with 58% grade 3 at baseline before treatment. Approximately 85.7% of men with EF adequate for intercourse at presentation without medications were still potent either with or without medication at last follow-up (Table 3; Figure 6).

## Discussion

Our study provides an updated comparison of FFBF and toxicity of SBRT and IMRT for the treatment of localized prostate cancer. To date, one other study has made this comparison, only reporting acute/chronic toxicity and cost comparisons between the two modalities in a national database (6). Our study offers late toxicity in addition to estimating FFBF with median follow-up times approaching 5 years. We also provided FFBF stratified by the 2015 NCCN guidelines of five risk groups which is lacking in the literature and the only factor significant in multivariate analysis for our study. We explored various known pretreatment factors in univariate analysis with other exploratory pretreatment factors such as race and age which we had not reported in our previous published experience (7, 9). We also explored use of hormone therapy as a confounding treatment variable in addition to fractionation scheme.

Intensity-modulated radiation therapy continues to be a standard radiotherapeutic technique for the definitive treatment of localized prostate cancer using a conventionally fractionated approach with 1.8–2.0 Gy fractions 5 days/week over 8–9 weeks. A decade of data supports this fractionation scheme for localized prostate cancer, with excellent biochemical control, overall survival, and acceptable toxicity (1). Phase 3 clinical trials of IMRT suggested that this new technology provided non-inferior control with acceptable acute toxicity, based on the rationale that IMRT was an innovation in targeting but prescription dose was based on an already proven fractionation scheme (10, 11). Because of an improved therapeutic ratio, dose escalation trials could be done with improved outcome (1, 12–15). Michalski et al. in 2013 reported the first randomized data showing a decrease in grade 2 and above GI toxicity from 22% with 3D-CRT to 15.1% with IMRT in the 79.2 Gy arm of RTOG 0126 (*p* = 0.04). In addition, although not statistically significant, grade 3 and above toxicity was reduced from 5 to 2.6% with IMRT (16). Updated analysis of this trial showed a decrease in biochemical failure, distant metastases with high dose 79.2 Gy compared with 70.2 Gy; however, no increase in survival was noted at 10 years with higher time to late grade 3 or greater GI toxicty for the 79.2Gy arm (17).

While we await results from an ongoing Swedish phase 3 trial of hypofractionated radiotherapy delivered in >5 fractions completed June 2015, there have been four reported phase 3 clinical trials of hypofractionation in >5 fractions compared with conventionally fractionated radiation which show no clinically significant difference in rates of efficacy and late toxicity, with median follow-up from 51 to 90 months (18–22). A recent non-inferiority-designed randomized controlled trial concluded no difference in efficacy with minimal increase in toxicity with a moderate hypofractionated regimen (23).

We also await the results of RTOG 0938, an equivalency study of low-risk prostate cancer, randomizing extreme hypofractionation with 36.25 Gy in 5 fractions compared with moderate hypofractionation of 51.6 Gy in 12 fractions. The study was closed February 2014 with 255 patients accrued with the primary objective of equivalence of 1-year health-related quality of life (24). No difference in 1-year quality of life or toxicity was recently reported in abstract form of this trial (5).

Several recent SBRT publications report non-inferior clinical efficacy with minimal toxicity. King et al. reported a prospective study including 1100 patients from 8 institutions which included 135 patients with at least 5-year follow-up. Among these 135, biochemical relapse-free survival rates were 99 and 93% for low- and intermediate-risk, with toxicity rates comparable to IMRT (4). While clinicians would like to see 10-year follow-up prospective randomized clinical trials to assess the efficacy of SBRT in the treatment of localized prostate cancer, the studies of SBRT that are available show encouraging results with follow-up approaching the single institutional trials of IMRT (1). Single institutions and pooled institutions or registries have shown efficacy and acute toxicity on par with IMRT (2–4, 25–27). A recent abstract by Katz reports the longest follow-up and outcome for a group of 515 men with organ confined prostate cancer stratified by three risk groups. With a median follow-up (FU) of 84 months, 9-year FFBF of 95% low risk, 89% intermediate risk, and 66% high risk groups was reported with 1.9% RTOG grade 3 urinary toxicity but no grade 3 GI toxicity (28).

With the 2015 NCCN risk stratification, multiple risk factors (which include Gleason score, pretreatment PSA, and clinical stage) promote stage migration and more homogeneity within groups. There were no biochemical failures for the very low-risk group within either SBRT or IMRT questioning the need for treatment in this very low-risk group. While biochemical failure was experienced in each other NCCN subgroup, the high and very high groups were the most at risk. Of interest is that despite no adjuvant lymph node treatment in the SBRT group, there was no significant difference in FFBR between SBRT or IMRT consistent with Lawton et al. and Amini et al. for high risk prostate cancer patients where no survival difference was noted between men treated with whole pelvis vs. prostate only conventionally fractionated external beam radiation (29, 30).

Stereotactic body radiation therapy seems well suited for prostate cancer for a number of reasons. The combination of prostate cancer’s low *a*/*b* ratio, demonstrated benefit of dose escalation above conventional doses, and efficacy/safety of high dose rate brachytherapy hypofractionation would lead one to believe SBRT should be at least comparable to IMRT in efficacy and toxicity for the treatment of prostate cancer. SBRT also has sparked interest due its convenience (duration of 1–2 weeks as compared with 8–9 weeks of IMRT treatment), non-invasive nature of treatment (compared with brachytherapy), and lower cost. Yu et al. recently published a study looking at Medicare records between patients treated with SBRT and IMRT and found that the mean treatment cost was $13,645 for SBRT vs. $21,023 for IMRT, at a time of increasing cost conscientiousness in the US (6).

Despite the fact all men were treated in a single hospital system within one radiation department, patients treated with SBRT and IMRT came from distinct treatment facilities with only one radiation platform available at each site with separate referral patterns. Low-risk patients were not preferentially selected for SBRT (Table 1). SBRT patients came from five different states, many self-referred and motivated for treatment with SBRT. IMRT patients alternatively came from the region surrounding the community hospital. Two physicians treated all patients with IMRT, while the majority of SBRT patients were treated by four physicians with only one physician treating at both sites. We began a prostate IMRT program in 2003 with significant experience by 2007 when this study began. Alternatively, we began an SBRT program in 2007 with early learning curve and more variation in treatment regimens in the early years. Of our SBRT patients who developed the most severe acute GU toxicity all were from the earliest era with higher doses and less experience. A recent dose escalation trial for prostate cancer treated with SBRT showed acceptable toxicities up to 47.5 Gy over 2.5 weeks (31). Our low toxicity in both the SBRT and IMRT groups suggest that there may be room for dose escalation with our series as well.

A commonly cited reason preventing widespread use of SBRT in localized prostate cancer is that adoption should not happen until we have results from randomized controlled phase 3 trials, due to fears of late toxicity. Yu et al. recently brought this issue to the forefront, with a comparison of SBRT to IMRT using Medicare beneficiary data on patients treated from 2008 to 2011. This study showed an increase in early and late GU toxicity with SBRT as compared with IMRT, with the respective increase at 6, 12, and 24 months in the rates of GU toxicity of 3% (15.6 vs. 12.6%), 3.9% (27.1 vs. 23.2%), and 7.6% (43.9 vs. 36.3%) (6). In contrast to the Yu study, we were able to assess baseline GU function and GU function status post SBRT and IMRT, in addition to grading the severity of GU toxicity. We found no difference between SBRT and IMRT in grade 2 GI and GU toxicity and no grade 3 toxicity after treatment in either group at most recent follow-up. Because we coded toxicity at last follow-up, the cumulative risk may be underestimated for patients with limited follow-up.

The limitations of this study include fewer high and very high risk men treated with SBRT and IMRT compared with the larger groups of very low-, low-, and intermediate-risk patients and limited power for multivariable assessment given the sample size and number of events. Another limitation is the uneven distribution in use of ADT between radiation treatment groups which could affect FFBF in particular for high and very high risk patients. Our treatment groups were unbalanced with regard to prognostic factors significant in univariate analysis such as Gleason Score and NCCN risk group. In multivariable analysis, however, only NCCN risk group was significant for FFBF but treatment group (IMRT or SBRT) was not significant in univariate or multivariable analysis. It is possible that there are additional factors not accounted for which may affect these results which is inherent in all retrospective analyses.

We did not routinely perform quality of life measurements prior to initiation of radiation with either IMRT or SBRT; however, we are now routinely obtaining Expanded Prostate Cancer Index Composite (EPIC), International Prostate Symptom Score (I-PSS), Bowel Health Inventory, and Sexual Health Inventory for Men (SHIM) in follow-up. Using patient-reported outcomes for RTOG 0126 trial, no difference was noted for the 79.2 Gy dose level between IMRT and 3-dimensional conformal radiation therapy through 24 months for bladder, bowel or erectile function. This study highlights the importance of patient reported outcomes as well as toxicity scales such as reported by the RTOG (32). The strength of this study is the uniform treatment for both IMRT and SBRT in a single hospital department with very separate referral patterns which limited treatment selection bias analyzed by known prognostic factors including stratification by NCCN 2015 guidelines.

In our experience, SBRT is an alternative to IMRT in the treatment of localized prostate cancer, with no significant difference between SBRT and IMRT for FFBF found. In this study, we validated the use of the 2015 NCCN guidelines for prostate cancer and have provided FFBF estimates using the five risk strata. To the best of our knowledge, no previous database describing outcome for prostate cancer following radiation has validated the NCCN risk strata, which is the only prognostic factor significant in multivariable analysis. Comparative effectiveness data are lacking for IMRT vs. SBRT for prostate cancer. Our paper contributes to this literature, which is hypothesis generating but awaits direct comparison in a randomized trial.

## Author Contributions

RL – treated patients in review, conceived project, data management, statistical review and analysis, manuscript preparation, review, and submission. AR – data management, statistical review and analysis, manuscript preparation, review and submission. GM – data management, analysis, statistics, and review of manuscript. AH – statistical analysis, manuscript preparation, and review. LB – treated patients in review, manuscript preparation, and review. JL – treated patients in review, manuscript preparation, and review. SA – treated patients in review, manuscript preparation, and review. MM – treated patients in review, manuscript preparation, and review. BG – treated patients in review, manuscript preparation, and review. JY – reviewed concept for research, supported physics analysis, manuscript preparation, and review. JF – supported physics analysis, manuscript preparation, and review.

## Conflict of Interest Statement

The authors declare that the research was conducted in the absence of any commercial or financial relationships that could be construed as a potential conflict of interest.

## Footnotes

^1^Only eight patients at risk with very high risk prostate cancer at 4 years.
